# Association of Anemia with Stroke Severity in Acute Ischemic Stroke Patients

**DOI:** 10.7759/cureus.2870

**Published:** 2018-06-23

**Authors:** Muhammad F Khan, Ibrahim Shamael, Qamar Zaman, Asad Mahmood, Maimoona Siddiqui

**Affiliations:** 1 Neuroscience, Shifa International Hospital, Islamabad, PAK; 2 Neurology, Shifa International Hospital, Islamabad, PAK; 3 Medicine, Queens Medical Center, Nottingham, GBR

**Keywords:** acute ischemic stroke, anemia, stroke severity, hemoglobin, islamabad, pakistan, nihss

## Abstract

Objective: To investigate the association of anemia with stroke severity in acute ischemic stroke patients.

Material & methods: We enrolled 96 patients (mean age: 60.25 ± 11.92 years old) who were admitted to the stroke unit of Shifa International Hospital between 1st March 2015 and 31st August 2015. Each patient presented within 72 hours of onset of symptoms, underwent computed tomography (CT) of the head and blood tests, including hemoglobin concentration, on the first day of hospitalization. Stroke severity was assessed on admission using the National Institute of Health Stroke Scale. Anemia was evaluated according to the World Health Organization (WHO) criteria (men, <13 g/dL; women, <12 g/dL). We examined the frequency of anemia in patients with different severities of acute ischemic stroke.

Results: World Health Organization defined anemia was positive in 38 (39.6%) and negative in 58 (60.4%) patients. Among the patients who were positive for anemia, seven (18.4%) had a minor stroke, 10 (26.3%) had a moderately severe stroke, and 21 (55.3%) had a severe stroke. There was a significant association between anemia and stroke severity (P-value 0.000).

Conclusion: Our data indicated that anemia was a frequent finding in acute ischemic stroke patients, with increasing frequency corresponding to stroke severity.

## Introduction

Anemia is a major health issue and a common condition among older adults, with its prevalence increasing with increasing age [1]. It is frequently associated with hospitalization, disability, and mortality [2]. Adverse outcomes, and poor survival too, have been associated with anemia, which has come into view as a risk factor. In an acute ischemic stroke (AIS) patient, anemia is associated with a poor prognosis [3-4].

The exact relationship between hemoglobin levels and outcome after an ischemic stroke is not completely understood. It is believed that extremes of both low and high hemoglobin are associated with a poor outcome. One of every five patients presenting with an ischemic stroke has anemia, and it is associated with a poor neurological outcome [1].

The global burden of stroke has the largest contribution from Asia, as the incidence of stroke here is higher than in Western countries [5]. Stroke has imposed an immense burden on society in the form of emotional, financial, and functional loss that is very hard to estimate [6]. It is a major cause of worldwide morbidity and mortality [7]. In comparison to other countries, stroke prevalence in Pakistan is one of the highest in the world. A community-based study has shown the crude prevalence of stroke to be 19,000 per 100,000 [8].

In Pakistan, stroke and anemia both constitute a major healthcare concern [8-9]. Currently, there is insufficient literature available in Pakistan regarding the association of anemia and AIS. The impact of anemia in ischemic stroke patients is unjustifiably underestimated [10]. The rationale of this study was to highlight the frequency of anemia in relation to stroke severity. The outcome would provide us a better understanding of the stroke burden, which may help in the effective management of patients, leading to improved healthcare planning and resource allocation. We designed this study to investigate the association of anemia with stroke severity in AIS.

## Materials and methods

A cross-sectional descriptive study was conducted on patients diagnosed with AIS who were hospitalized at Shifa International Hospital (SIH), Islamabad, from 1st March 2015 to 30th August 2015.

Data was collected through consecutive nonprobability convenience sampling. We used the online World Health Organization (WHO) calculator, with a 95% confidence interval, a population proportion of 14.2%, and a required absolute precision of 7% to calculate the sample size, which was estimated to be 96.

Patients with ages ranging from 18 to 70 years and presenting within 72 hours of onset of symptoms were included. Patients with transient ischemic attacks, hemorrhagic stroke, history of a previous stroke, venous stroke secondary to dural sinus thrombosis, concomitant acute coronary syndrome, and an underlying disability due to any other cause were excluded.

**Figure 1:**
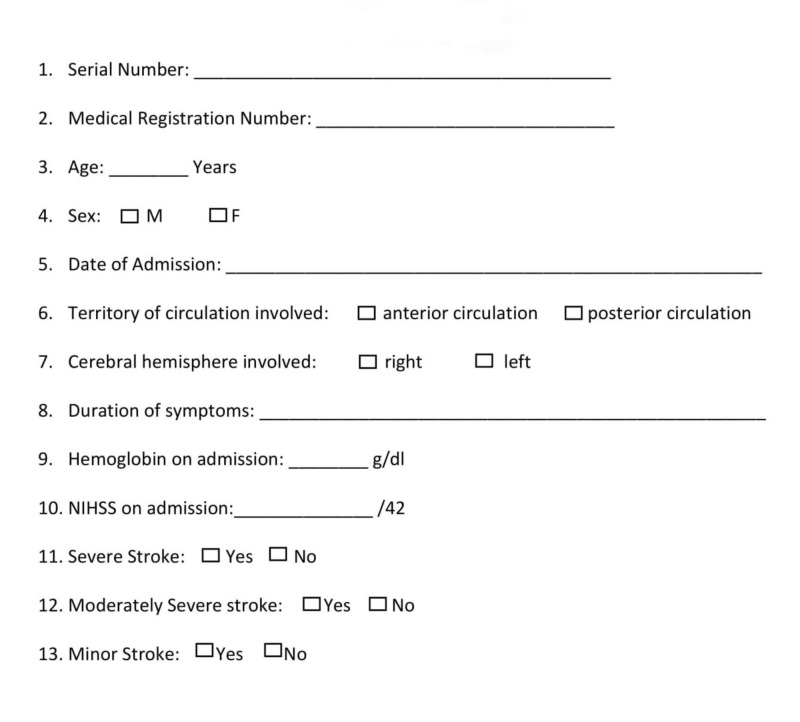
Medical data collection form NIHSS: National Institute of Health Stroke Scale

Written informed consent was obtained from all participants fulfilling the inclusion criteria. AIS was confirmed by the results of the baseline head CT scan. A personal interview and a clinical evaluation were conducted on all patients. Each patient’s information was entered in a predesigned medical data collection form (Figure 1).

The severity of stroke was assessed using the National Institutes of Health Stroke Scale (NIHSS) [11].

Scores of 16 or greater, 9-15, and 1-8 were labeled as severe stroke, moderately severe stroke, and minor stroke, respectively [12-13].

Patient’s hemoglobin levels were checked by the Shifa International Hospital (SIH) hematology laboratory and verified by a certified pathologist. Hemoglobin was measured with the Sysmex Hematology Analyzer XE-5000 (Sysmex Corporation, Kobe, Japan) using the manufacturer's reagents and methods. Anemia was evaluated according to the WHO criteria (men, <13 g/dL; women, <12 g/dL) [14].

Ethical approval of this study was received from the SIH Institutional Review Board and Ethics Committee.

Data analysis procedure

Data were entered and analyzed using statistical package for social sciences (SPSS) version 21 (IBM, Armonk, NY, US). For continuous variables like age, mean and standard deviation were applied. For categorical variables like gender, anemia, and stroke, severity frequencies and percentages were calculated. Effect modifiers like age and gender were controlled by stratification. Post-stratification, the Chi-square test was applied. A P-value of less than or equal to 0.05 was considered statistically significant.

## Results

A total of 96 patients with AIS were included in the study. The mean age was 60.25 ± S.D 11.93 years, with an age range of 21-70 years. The study population comprised 49 (51%) males and 47 (49%) females.

Out of the 96 patients, three (3.1%) were in the age group of 15-25 years, two (2.1%) were in the age group of 26-35 years, 10 (10.4%) were in the age group of 36-45 years, five (5.2%) were in the age group of 46-55 years, 40 (41.6%) were in the age group of 56-65 years, and 36 (37.5%) were in the age group of greater than 65 years.

**Figure 2:**
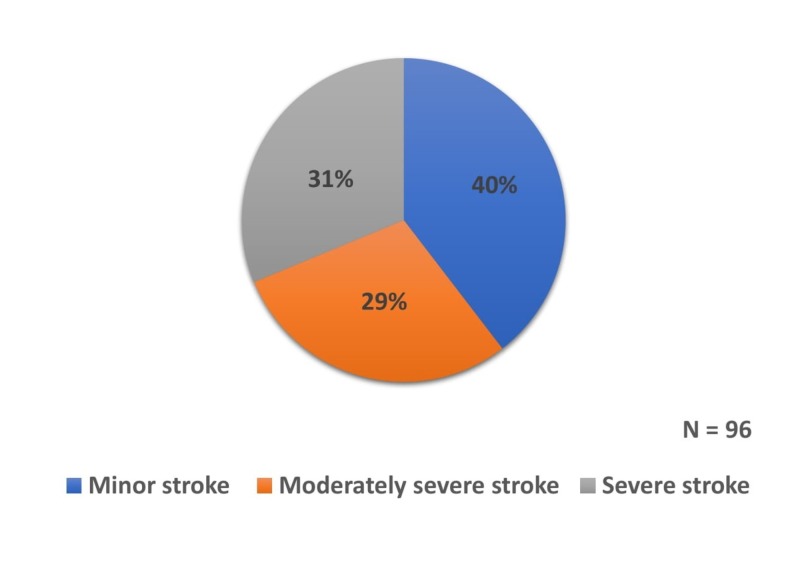
Distribution of patients based on severity of stroke

The distribution of severity of stroke in our study is shown in Figure 2.

WHO-defined anemia was positive in 38 (39.6%) and negative in 58 (60.4%) patients. Among the 38 AIS patients who were found to be positive for anemia, seven (18.4%) had a minor stroke, 10 (26.3%) had a moderately severe stroke, and 21 (55.3%) had a severe stroke.

The stratification of patients with anemia was done in terms of severity of stroke (Table 1).

**Table 1:** Stratification of anemia based on severity of stroke

Anemia	Stroke severity			Total	P-value
	Minor stroke	Moderately severe stroke	Severe stroke		
Positive	7	10	21	38	0.000
Negative	31	18	9	58	
Total	38	28	30	96	

The Chi-square test revealed a P-value of 0.000, hence suggesting a statistically significant association between the two variables.

Out of 38 patients in the anemic category, there were 14 males and 24 females. Out of 58 patients in the non-anemic category, there were 35 males and 23 females. Anemia was positive in more female patients as compared to their male counterparts. The relative frequencies of both genders are shown in Table 2.

**Table 2:** Stratification of anemia based on gender

Anemia	Gender		Total	P-value
	Male	Female		
Positive	14	24	38	0.024
Negative	35	23	58	
Total	49	47	96	

The Chi-square test showed a P-value of 0.024, suggesting a statistically significant difference between the two genders based on anemia.

A comparison of various age groups based on the presence of anemia showed no significant difference as the Chi-square test showed a P-value more than 0.05.

## Discussion

The global anemia prevalence is estimated to be 32.9%, with South Asia being included among the regions with the highest burden. Its overall prevalence in females is higher in most regions and age groups [15].

Anemia is a major problem faced by a developing country like Pakistan [9]. Our study showed that the female gender was more anemic and more than half of the participants (55.3%) who tested positive for anemia had a severe stroke.

Low socioeconomic conditions, the consumption of cereal-based diets with a low bioavailability of iron, high consumption of cereals, legumes, and plant-based diets, inappropriate personal hygiene, early marriage, repeated pregnancies, and low literacy rate are the major factors causing anemia in the Pakistani population [16].

Other studies, associating low hemoglobin levels with poor outcome in AIS patients, are mostly the ones in which hemoglobin testing was done on admission only. However, the continued monitoring of hemoglobin levels during the hospital stay of the patient may have more significance to the outcome than the baseline admission level. Limiting blood sampling to the necessary minimum, avoiding fluid overload, preventing and treating infections early, and controlling kidney function are reasonable interventions once anemia is diagnosed in an AIS patient [10].

Our study results show that anemia could lead to significant morbidity and disability in AIS patients. However, more evidence and further research are required to shed light on the management of anemic patients with AIS.

The clinical implications of our study cannot be implemented right away. There were certain limitations in our study. This was a hospital-based study with a small sample size. We did not categorize anemia according to the severity or cause, nor did we differentiate different types of anemia. In order to label anemia as a critical prognostic factor, a population-based study on a larger scale is required, which will give us more accurate results. Research is needed on the pathophysiology, forms, causes, and treatment modalities of anemia in patients with AIS.

## Conclusions

We conclude anemia is a frequent finding in patients with AIS. Its frequency is seen to increase with an increase in stroke severity. Healthcare providers treating ischemic stroke patients should be more meticulous in its early identification and treatment.
